# Limited beneficial effects of systemic steroids when added to standard of care treatment of seasonal allergic rhinitis

**DOI:** 10.1038/s41598-023-46869-4

**Published:** 2023-11-10

**Authors:** Carl Skröder, Laila Hellkvist, Åslög Dahl, Ulla Westin, Leif Bjermer, Agneta Karlsson, Lars Olaf Cardell

**Affiliations:** 1 Department of Otorhinolaryngology, Head and Neck Surgery, Skane University Hospital, Lund, Sweden; 2 Division of ENT Diseases, Department of Clinical Sciences, Intervention and Technology, Karolinska Institutet, Stockholm, Sweden; 3 Department of ENT Diseases, Karolinska University Hospital, Stockholm, Sweden; 4 Departments of Biological and Environmental Sciences, Gothenburg University, Gothenburg, Sweden; 5 Department of Otorhinolaryngology, Head and Neck Surgery, Institute of Clinical Sciences, Skane University Hospital, Lund, Sweden; 6 Department of Respiratory Medicine and Allergology, Lund University, Skane University Hospital, Lund, Sweden

**Keywords:** Randomized controlled trials, Chronic inflammation

## Abstract

Intramuscular injections with methylprednisolone treating allergic rhinitis (AR) have a long history. Modern guidelines are designed to dissuade this treatment, but it´s frequently used, especially in primary care. This despite of concern for side effects and lack of modern placebo-controlled studies. This study was designed to evaluate if methylprednisolone, could significantly improve symptoms of birch pollen induced AR and reduce the concomitant use of standard of care medication. Forty-two patients with birch pollen induced AR were randomized to treatment with methylprednisolone (80 mg) or placebo (NaCl 0.9%). Daily symptom- and medication scores was registered for 3 weeks. Quality of life questionnaires Sino-nasal Outcome Test-22 (SNOT-22) and Juniper Rhinoconjunctivitis Quality of Life Questionaire (Juniper RQLQ) were registered at trial start and at the end of the 3 weeks period. The combined symptom- and medication scores indicate that the methylprednisolone treated group [mean Area Under the Curve (AUC) 37.1 (SD 16.2 (95% CI 29.9–44.6))] was significantly better off than the placebo group [mean AUC 49.1 (SD 10.1 (95% CI 44.5–53.7))], p = 0.008. No significant difference between the groups were found in the SNOT-22 and Juniper RQLQ analysis. Registered side effects were few and mild. The limited beneficial effects of systemic steroids when added to standard of care in combination of its potential risk for side effects, speaks against its use for treatment of severe seasonal allergic rhinitis. The lack of difference in quality-of-life further underscores this result.

## Introduction

Allergic rhinitis (AR) is a chronic condition with a 30% prevalence in Sweden^1^. Typical AR symptoms include rhinorrhea, nasal obstruction or blockage, nasal itching, sneezing, and postnasal drip. ^2^. It is also often associated with itching and redness of the eyes and a severe tiredness. The later known to affect both work and performance at school. Despite widespread availability and frequent use of standard of care medication the majority of patients are unsatisfied and report a marked impairment in their quality of life^3^. The high prevalence of AR and lack of satisfactory treatment led to loss in productivity (presenteeism) resulting in high costs for the society^4,5^.

For symptoms progressing from mild/intermittent to severe/persistent, rhinitis patients should first be treated with oral antihistamines and/or intranasal steroids. Immunotherapy should be considered from moderate/intermittent and mild/persistent symptoms through to severe/persistent symptoms. When this fails, like in the middle of a severe pollen season, short-term systemic corticosteroids are often prescribed, especially in primary care. The use of oral corticosteroids is recommended, but not scientifically supported, in modern guidelines. Intramuscular corticosteroid injections are not recommended due to the risk of side effects^2,6^. Despite this, the longstanding praxis of giving a pre-seasonal intramuscular injection methylprednisolone remains at several places around the world.

The occurrence and severity of corticosteroid side effects are seen to depend upon the duration of use, dosage, dosing regime and specific drug used, along with individual patient variability^7^ . Short-term steroids are used, especially in general practice, for a variety of reasons. Not only seasonal allergic rhinitis and asthma, but also for upper respiratory infection, spine conditions, acute bronchitis, connective tissue and joint disorders and skin disorders^8^. Prescribing oral corticosteroids in short courses may seem to be free from significant adverse effects. But according to data derived from private insurance claims, involving 1.5 million people, significantly higher rates of sepsis, venous thromboembolism and fractures are found among the steroid treated population, even with a relatively brief duration of treatment^8^. Using short courses of corticosteroids in high doses as treatment of asthma, has shown to accumulate higher doses compared to maintenance treatment, which may be prejudicial to health^9^.

We found only two studies that assessed the efficacy of systemic steroids for allergic rhinitis performed during the last 30 years. The first is a comparison of various doses of oral methylprednisolone, three times a day, during the first 4 days of the rag weed season, published 1993 ^10^. The second is an open labeled study from 2013 that compared intranasal steroid spray (mometasone furoate) with betamethasone oral tablets. So far there is no other modern study that evaluates the potential effects of systemic steroids on allergic rhinitis ^11^. The present study is a double blinded, placebo-controlled study of methylprednisolone injections given at the start of the birch pollen season in Sweden.

## Methods

### Study design

**Figure 1:**
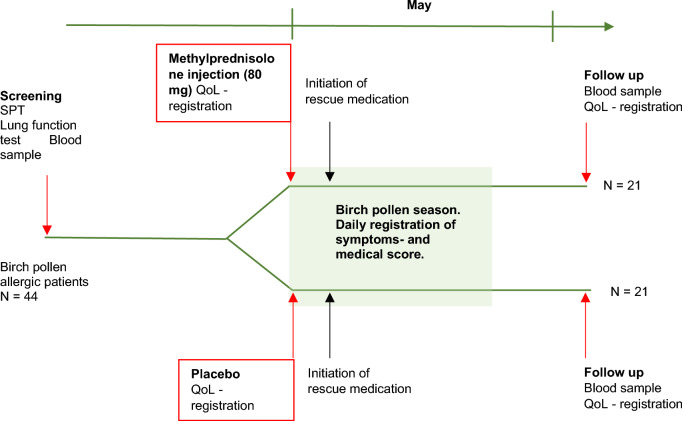
Study outline.

This was a single-center, double-blinded, randomized, placebo-controlled trial treating two groups in parallel. The study was performed over 3 weeks starting just prior to the birch pollen peak of the season in April to May 2019 (Fig. 1).

The study was approved by the National Research Ethics Committee and was conducted according to good clinical practice guidelines. All methods were performed in accordance with national guidelines and regulations as well as the Declaration of Helsinki. All participants provided written informed consent. The trial was registered (04/12/2018) in the EudraCT database as no. 2018-004205-12 and in the register of ClinicalTrials.gov (first posted 10/11/2020 and last release 24/06/22) with identifier NCT04622917. All data generated or analyzed during this study are included in this published article (se supplementary information files).

### Study subjects

Patients included were in the age 18–40 years, had a history of seasonal moderate to severe pollen induced allergic rhinitis during birch season according to the Allergic Rhinitis and its Impact on Asthma classification^2^.

### Allocation to treatment and blinding

Allocation to treatment arms were aided by a computer randomization program in blocks.

### Pollen data

Pollen data were registered with a Burkard 7-day Volumetric Spore Trap (Fig. 5). Pollen concentrations is registered in supplement Fig. 2.

### Treatment protocol

Patients received either an intramuscular injection methylprednisolone 80 mg as a single dose or an intramuscular injection with saline solution (NaCl 0.9%, B.Brown). Injections were given during a period of 6 days. All patients received their injections before the pollen peak of the season. Pre-trial all patients received a “Rescue medication package” (containing Desloratadine tablet 5 mg × 1, sodium cromoglycate eye drops 40 mg/ml 1–2 drops × 2, Mometasone Furoate nasal spray 50 μm × 2). The rescue medication was not allowed after trial start until Day 3 after 2 consecutive days of symptoms and could then be used throughout the trial.

### Outcome measures

#### Primary outcome measure

Improvement of symptoms and reduced use of standard of care medication in patients treated with methylprednisolone compared to placebo. The daily symptoms and medications used during the study period were registered according to European Academy of Allergic and Clinical Immunologys (EAACI:s) recommended scoring system ^12^. The patients were instructed to use rescue medications stepwise. The diary and questionnaires were sent by e-mail, using the software RedCap.

A daily symptom score(dSS), daily medication score (dMS) and daily combined symptom- and medication score) dCSMS were expressed as the median value of each group every day of the study period. The median value was used because of the limited sample size. Minimal clinical important difference (MCID) has not been defined for combined symptom- and medication score. We estimated a significant MCID to about 20% ^13^.

#### Secondary outcome measures

Quality of life (QoL) was assessed with the questionnaires Sinonasal outcome Test -22 (SNOT-22) and Juniper Rhinoconjunctivitis Quality of Life (Juniper RQLQ). SNOT-22 has 22 questions with a score between 0 and 5. Shamim Toma and Clair Hopkins has provided a suggestion subdividing the total SNOT-22 score into mild 8–20, moderate > 20–50, and severe > 50 ^14^. SNOT-22 was developed to analyze chronic rhinosinusitis. Efforts of evaluating SNOT-22 as a tool of analyzing AR has been made with promising results, but uncertainty regarding minimal clinical different score, ranging between 6 and 11 ^15^. Juniper RQLQ consists of 28 questions, subdivided into seven sections, with an individual score for each question between 0 and 6. The score of each question was valued equally and the mean of the total score was calculated. Minimal clinical important difference is 0.5.

### Spirometry

A spirometry (Jaeger®) was performed on all participants before the trial.

### Skin prick test

SPT was performed on all patients using ALK Soluprick^®^ SQ.

### Blood measurements

Venous blood samples were obtained at the screening visit and four to five weeks after the methylprednisolone/placebo injection.

### Statistical analysis

dSS, dMS and dCSMS were expressed as the area under the curve (AUC) and the differences between the groups were calculated using a Mann–Whitney test. A Wilcoxon matched-pairs signed rank test was performed on SNOT-22 and Juniper RQLQ when comparing day 1–21. A Mann–Whitney test was used to analyze the individual difference day 1–21 between the groups.

In a time-series analysis a generalized additive model (GAM) was used to evaluate the relative risk (RR) for symptoms in the study group.

### Sample size

Using a two-sample t-test based on expected improvement on total nasal symptoms score (TNSS) a power calculation was performed. Assuming a 2 out of 12 points improvement in total dSS and a standard deviation of 2 points, aimed at a power of 0.80 and using a type 1 error rate α level of 0.05, the calculated sample size was 32. A power calculation using CSMS was not performed prior to study start. Patients treated with Depomedrol had a median (IQR) CSMS score of 1.6 (1.3–2.4) and in the placebo group 2.5 (1.8–3.0).

## Results

### Patients

**Figure 2:**
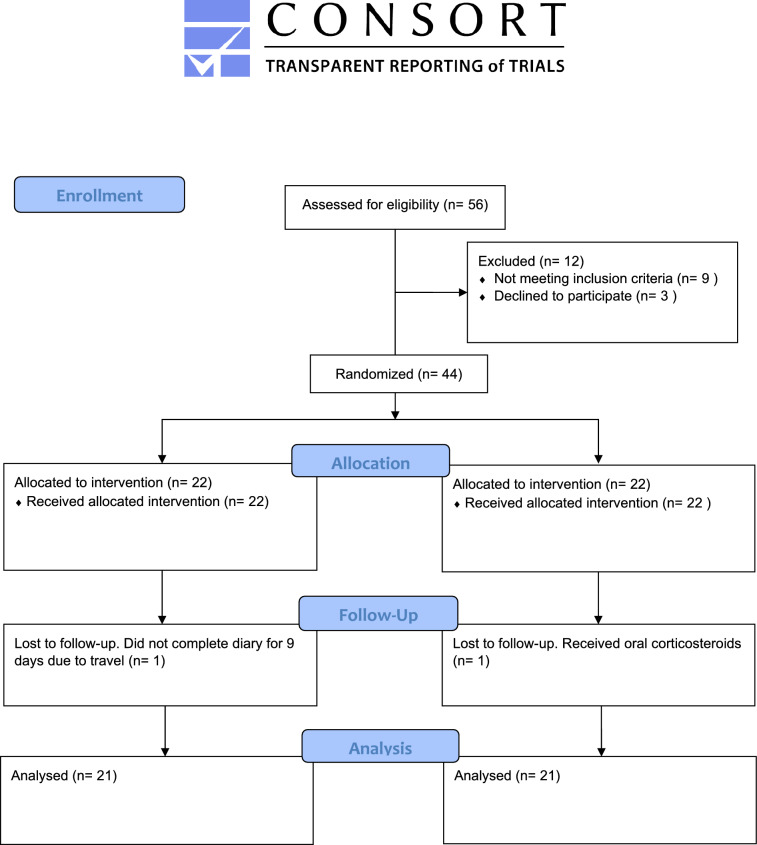
CONSORT 2010 flow diagram.

As seen in Fig. 2, forty-four patients were randomized and given treatment/placebo in two equally sized groups. Baseline characteristics of the study population did not differ significantly between the groups, as seen in Table 1. The response rate answering the e-diary and QoL forms was similar in the treated group (n = 21, 92%) and the placebo group (n = 21, > 94%). Missing data of 6 days or less for each patient was accepted in the statistical analysis, except for calculating the AUC.

**Table 1:** Background characteristics.

	Placebo group	Active group	P-value
Number of patients	21	21	
Gender			
Female, no. (%)	11 (52%)	9 (43%)	
Male, no. (%)	10 (48%)	12 (57%)	
Age, mean [SD (range)]	41 [9 (24–55)]	37 [9 (20–50)]	0.24
Sensitization to birch pollen on SPT, no. (%)	21 (100%)	22 (100%)	
Birch specific IgE (kU/L), median [SD (range)]	15 [13 (3–55)]	17 [14 (0.7–62)]	0.82
Other allergy			
House dust mite, no. (%)	9 (43%)	11 (52%)	
Gras, no. (%)	12 (57%)	19 (90%)	
Mugwort, no. (%)	8 (38%)	11 (52%)	
Furry animals, no. (%)	12 (57%)	11(52%)	
Verified seasonal asthma, no. (%)	2 (10%)	1 (5%)	
Use of beta-2-agonist, no (%)	7 (33%)	5 (24%)	
Use of inhaled corticosteroids, no (%)	5 (24%)	3 (14%)	

### Symptom and medication scores

**Figure 3:**
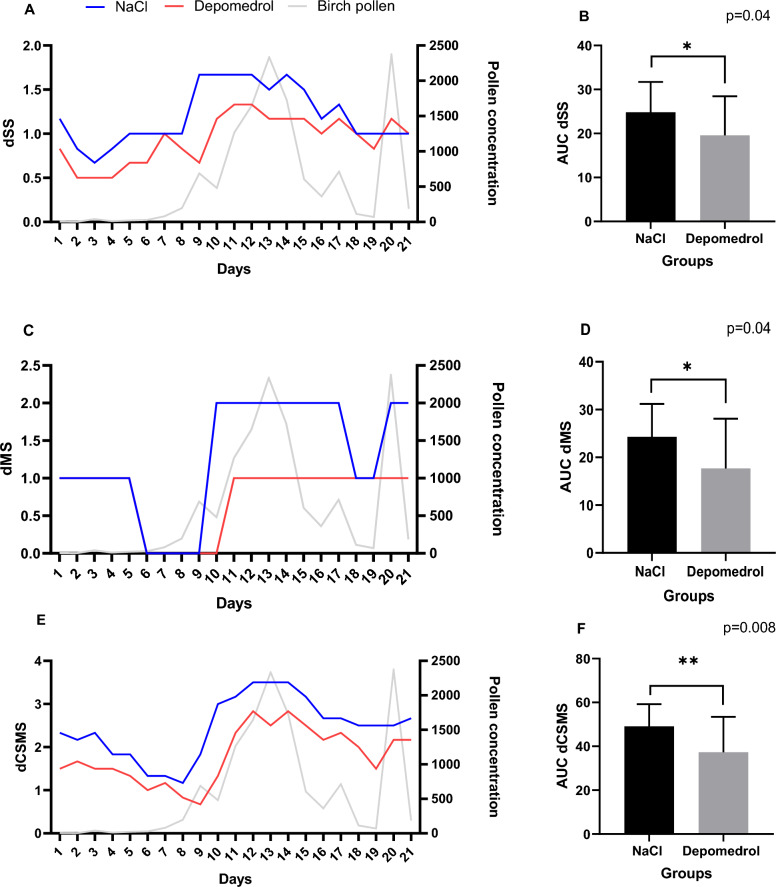
(**A,C,E**) The daily median score (dSS, dMS and dCSMS) in each group from day 1–21. (**B,D,F**) The mean of total AUC (dSS, dMS and sCSMS) in each group. p-value from Mann–Whitney test.

There was a difference in dSS, dMS and dCSMS between the groups throughout the birch pollen season 2019. The treated group experienced less symptoms with mean AUC 19.6 [SD 8.8 (95% CI 15.6–23.6)] compared to the placebo group with mean AUC 24.8 [SD 6.9 (95% CI 21.7–28.0)], p = 0.04. The treated group used less rescue medication with mean AUC 17.7 [SD 10.4 (95% CI 12.9–22.4)] compared to mean AUC 24.3 [SD 6.9 (95% CI 21.1–27.4)] in the placebo group, p = 0.04. The combined symptom and medical score mean AUC 37.1 [SD 16.2 (95% CI 29.9–44.6)] in the treated group was approximately 24% lower than the mean AUC 49.1 [SD 10.1 (95% CI 44.5–53.7)] in the placebo group, p = 0.008 (Fig. 3).

### Quality of life

There was no significant difference in quality of life in neither group comparing, both mean- and total scores at trial start and after three weeks. Mean SNOT-22 in the placebo group was 1.6 [SD 1.1 (95%CI 1.1–2.1)] day 1 and 1.6 [SD 1.0 (95%CI 1.1–2.0)] day 21, p = 0.98. The treated group had a mean SNOT-22 1.0 [SD 0.6 (95% CI 0.7–1.3)] day 1 and 1.3 [SD 0.8 (95% CI 1.0–1.7)] day 21, p = 0.08. Both the mean total SNOT-22 score in the placebo group (35.3 at the injection day and 34.7 after 3 weeks) as well as the methylprednisolone group (22.6 at the injection day and 29.3 after 3 weeks) were moderate ^14^.

Mean Juniper RQLQ in the placebo group was 2.2 [SD 1.2 (95% CI 1.6–2.7)] day 1 and 2.2 [SD 1.3 (95% CI 1.6–2.8)] day 21, p = 0.75. The mean Juniper RQLQ in the treated group was 1.5 [SD 1.0 (95%CI 1.1–2.0)] day 1 and 1.9 [SD 1.1 (95% CI 1.4–2.4)] day 21, p = 0.22.

**Figure 4:**
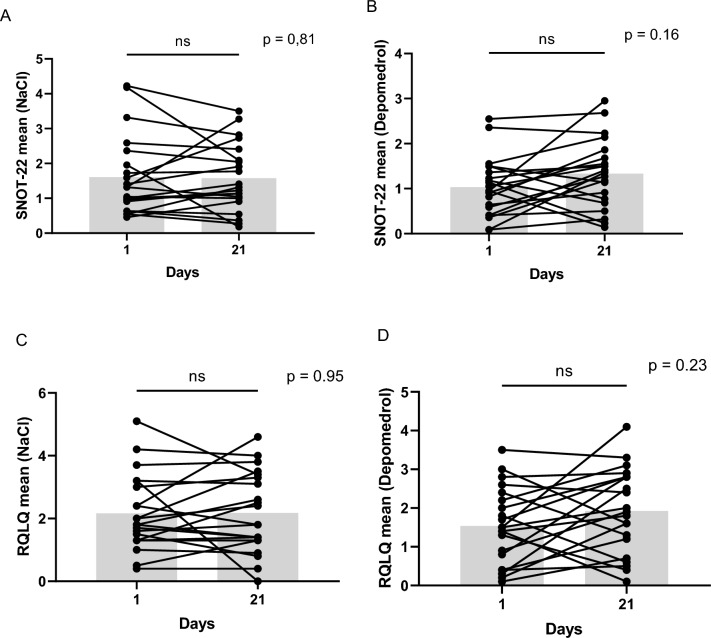
**(A,B)** Individual changes in SNOT-22 in each group. (**C,D**) Individual changes in Juniper RQLQ in each group. Greay boxes represents median SNOT-22 and Juniper RQLQ. The lines represent the individual change registered at day 1 and 21.

When comparing mean SNOT-22 in the placebo group versus the treated group day 21 no significant difference could be seen, p = 0.6 or when comparing the difference between day 1–21, p = 0.2. No significant difference was seen comparing Juniper RQLQ in the placebo group and the treated group day 21, p = 0.6 or comparing the difference between day 1–21, p = 0.29 (Fig. 4).

### Pollen data

**Figure 5:**
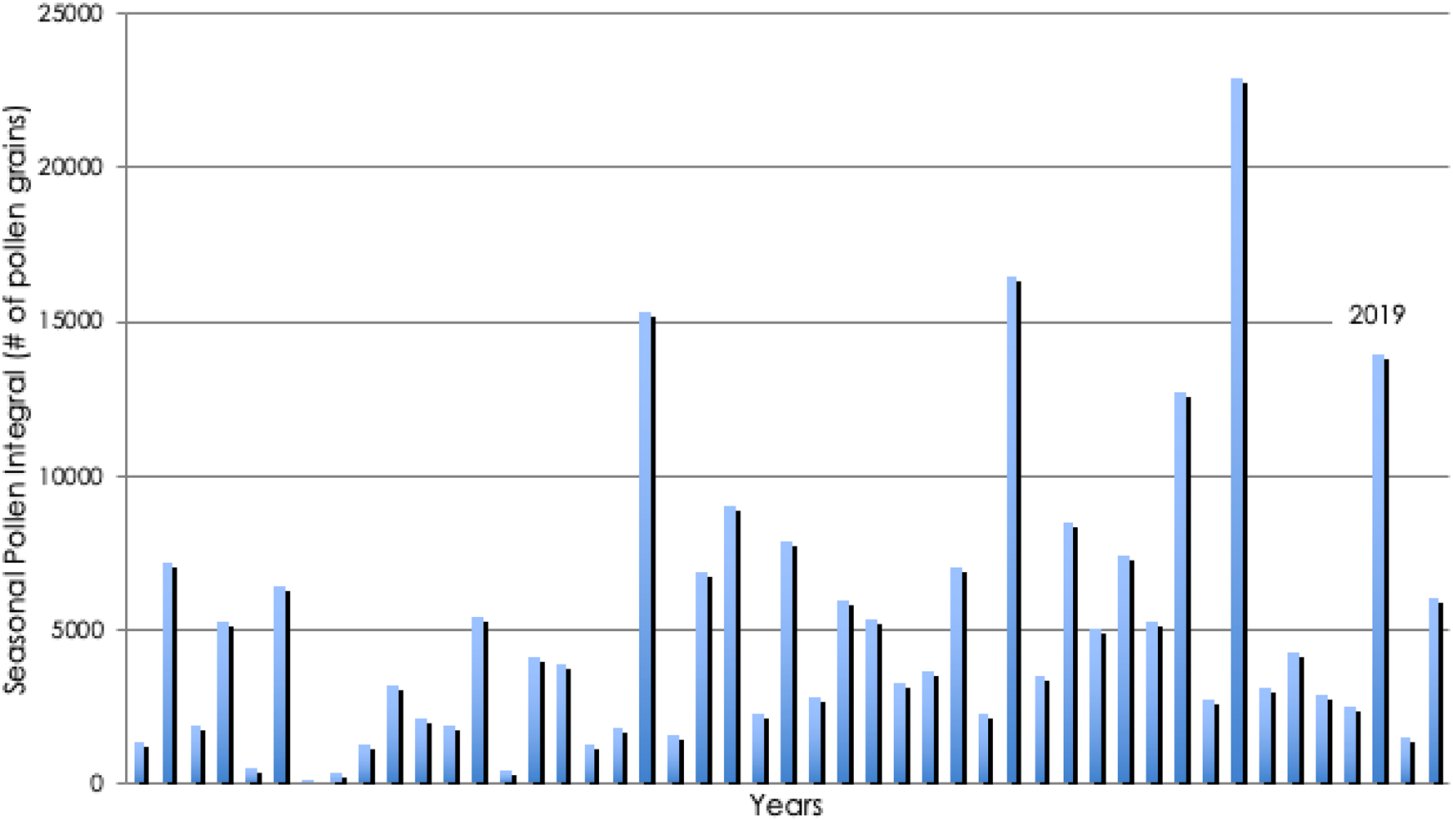
Airborne birch pollen concentrations in Malmo 1975–2021.

The Seasonal Pollen Integral of birch pollen in Malmö 2019 (13,948 pollen) was the 4th highest since the start of the pollen monitoring at this site in 1975, and 260% of the average value, considerably higher than during the previous four years (see Fig. 5). The relative risk (RR) for symptoms caused by birch pollen in the study group is seen in supplement Fig. 1.

### Safety

Adverse events occurred in both groups and were mild. Patients expressed symptoms such as nasal blockage, nasal drip, sneezing, and difficulty breathing while exercising, in both groups. One patient in the placebo group had a severe headache, another received a rash on the stomach and back and experienced trouble falling asleep. The symptoms resolved within a day. In the treated group two patients expressed problems with nosebleed, one became sensitive to light, another experienced trouble falling asleep and had nightmares and one got aphthous ulcers. The symptoms resolved within one to three days. No SAE were reported during the study period.

All patients where within the reference interval both before- and after trial start when analysing ACTH and bone turnover marker CTx (see supplement Tables 1 and 2). The blood sample indicate short term degradation of bone minerals which may lead to osteopenia or osteoporosis in the long run.

## Discussion

The present study demonstrates that a single injection of methylprednisolone in conjunction with the start of a birch pollen season, characterized by a severe increase in the amount of airborne pollen, reduced nasal and eye symptoms and resulted in a less frequent use of rescue medication than placebo. The symptom reducing effect was statistically significant, but surprisingly small. There was a 24% improvement in AUC of dCSMS between the two groups. The World Allergy Organization (WAO) recommend an 20% reduction in CSMS when conducting AIT trials to conclude a significant improvement compared to regular antihistamine treatment ^13^. Advocates for systemic steroids treating AR often describes a satisfied patient with no need of additional treatment. If this was the case an improvement for example at least 50% would be expected. No systemic steroid induced improvement in quality of life was seen. The difference of dCSMS between active treatment and placebo was highly significant but so small that the needed number of participants is believed to be increased multiple times to reflect a significant difference in comparison of QoL.The mean total SNOT-22 score was moderate in both groups according to the grading system proposed by Toma and colleagues and did not differ significantly in neither of the groups ^14^. The result must be interpreted with caution since the use of SNOT-22 in regards of AR is more uncertain than for example evaluation of chronic rhinosinusitis treatment. The reported short term side effects were few and mild. In Sweden pre-seasonal intramuscular injected Methylprednisolone 80 mg, as a single dose, has been a standard, addon treatment when topical treatment doesn´t reduce AR symptoms sufficiently. Our choice of dosage regime and timing of treatment is in line with previous published data ^16^.

A problem with all studies of seasonal allergic rhinitis is the variation of pollen exposure among the participants. During 2019 the Seasonal Pollen Integral, comprising all registered birch pollen during the season, was 13,940, which as 260% of the average value during the period 1975–2019 in the Malmö area, Sweden. This is considerably higher than during the previous four years (Fig. 5). The number of days with a pollen concentration exceeding the threshold values for high or very high levels, was also higher than average. It is therefore likely that all participants in the study were exposed to significant amounts of birch pollen.

The overall response rate among all the study participants was generally good (> 90%). During the baseline run in period, both groups reported dSS and dMS above 0, even though the birch pollen concentration during this period was low. The reasons for this could not be indubitably assessed but are most probably related to a lingering symptom response induced by pollen from birch related trees, like alder and hazel, with a flowering period in Sweden slightly prior to birch.

dCSMS has been proposed to be the goldstandard evaluation of treatment efficacy in allergic rhinitis ^12^.

In this study a modification of this scoring algorithm was necessary since systemic steroids were the focus of the evaluation. Despite this, it is our belief that our data is comparable to other clinical allergy trials using the original algorithm. The peak of the birch pollen season at day 9 coincided with the highest dCSMS levels both in the steroid and in the placebo group.

During 1960 to 1988, 18 clinical trials were performed investigating the effect of different injectable steroids treating seasonal allergic rhinitis in adults. Nine were double-blind (5 placebo-controlled and 4 comparative), two single-blinded, and seven open trials ^16^. The efficacy of a single intramuscular injection of steroid was statistically significant in all five placebo-controlled trials and demonstrated considerable clinical benefit, lasting approximately from within the first day to four weeks. In the two studies that compared intramuscular steroids with to nasal steroids a superior effect with intramuscular steroids was noted. In all studies, the side-effects were few, both clinically and physiologically. The ability to respond to stress with hypothalamic–pituitary–adrenal activation appeared to be retained, when evaluated ^16^. Even though some of these older investigations do not meet the methodological criteria of the scientific requirements of present-day studies, their homogenous message of a positive effect on nasal symptoms is striking. Their generally strong positive outcome stands in sharp contrast to the presently presented more indifferent effect. However, when it comes to reported short term side effects our study is well in agreement with the older reports.

In a more recent study, Brooks and colleagues compared the effect of three doses of oral methylprednisolone dosed three times a day (6, 12, or 24 mg a day), with placebo in 31 patients with ragweed hay fever during the first four days of the season. 24 mg daily showed statistically significant suppression of obstruction, running and sneezing. Itching showed a tendency to improvement, but it did not reach significance. The eye symptoms were improved at all doses. The treatment started the day before the start of symptom registration and lasted during the 4 days of registration, resulting in a total of 30, 60 and 120 mg, respectively given during the four-day long study period. This should be compared with the total of 80 mg methylprednisolone given as a single depot injection at the start of a three-week registration period in the present study. Thus, it is interesting to notice that middle dose of 60 mg total, which is most comparable to the 80 mg used in the present study, lacked effects on all nasal symptoms.

In the second of the only two studies that have investigated the effects of systemic steroids on allergic rhinitis during the last 30 years, the efficacy of intranasal steroid spray (mometasone furoate) was compared with oral corticosteroids (betamethasone) and placebo ^11^. In contrast to some^16,17^, but not all ^18^ older studies no significant differences were found in the therapeutic effects of the topical and systemic corticosteroids tested. The alleviation of symptoms was significantly better in the two steroid treated groups than in the placebo group. However, it is important to recognize that this was an open labeled study without placebo. The patients’ expectations might therefore have affected the outcome. Only mild side effects were noted in the steroid treated groups, with no difference in between.

Our primary endpoint was to investigate if a short course of corticosteroids conjures an effective treatment of AR. Short term safety aspects were determined using the secondary endpoints. During the pollen peak of the season the steroid treated group were less inclined to use nasal steroids but used antihistamine tablets and/or antihistamine eyedrops compared to patients in the control group who used all the available rescue medication. Even though symptom reduction was statistically significant and probably of some clinical value, it was much smaller than anticipated. This bleak effect was mirrored by the absence of steroid induced improvements in the quality of life.

No abnormal concentrations of ACTH or bone turn over markers were registered, and no SAEs were reported. These data are in line with the reports found in Swedish Medical Products Agency´s record for intramuscular injection methylprednisolone. Further, it corroborates the conclusion about mostly limited side effects made by Østergaard and colleagues, after reviewing 18 older studies of systemically injected steroids treating seasonal allergic rhinitis. It is important to notice that most publications only report on relatively short-termed side effects and that there is evidence for that the cumulative “life dose” of oral steroids can affect at least the long-termed risk for loss of bone mineral density and the coherent risk of fracture^19^. This must be considered when treating patients with intramuscular injections during several pollen seasons.

## Conclusion

Our findings conjure no strong evidence for the beneficial effects of using systemic steroids in addition to standard of care for treatment of seasonal allergic rhinitis during the peak of the pollen season. Hence, the use of intramuscular steroids in the treatment of seasonal allergic rhinitis must be questioned, not so much based on the risk of acute side effects, as for its limited efficacy. This was a limited sized study and further research is needed to conclude the result.

### Supplementary Information

Supplementary Information 1.

Supplementary Information 2.

Supplementary Figure 1.

Supplementary Figure 2.

Supplementary Tables.

## Data Availability

All data generated or analyzed during this study are included in this published article (and its supplementary information files).

## Abbreviations

AIT: Allergen immunotherapy
AR: Allergic rhinitis
SCIT: Subcutaneous immunotherapy
SLIT: Sublingual immunotherapy
RQLQ: Rhinitis quality of life questionnaire
SNOT-22: Sinonasal outcome test-22
TNSS: Total nasal symptom score
SPT: Skin prick test
QoL: Quality of life
dSS: Daily Symptome Score
dMS: Daily Medical Score
dCSMS: Daily Combined Symptom and Medical Score
MCID: Minimal clinical important difference
