# Supplementary material for: Limited beneficial effects of systemic steroids when added to standard of care treatment of seasonal allergic rhinitis
Source: Sci Rep. 2023 Nov 10;13:19649. doi: 10.1038/s41598-023-46869-4 (PMC10638382; doi:10.1038/s41598-023-46869-4)
Supplement: Supplementary file 1 — Supplementary Information 1. [file 41598_2023_46869_MOESM1_ESM.docx]

**Questions allergy diary**

**Estimate the discomfort during the last 24 hours due to a runny nose*** 0= No symptoms. 1=Mild symptoms (Symptoms are easily tolerated). 2= Moderate symptoms (Intense symptoms but tolerable). 3= Hard symptoms (Symptoms are hard to tolerate, effects daily activities and/or sleep).

- 0
- 1
- 2
- 3

**Estimate the discomfort during the last 24 hours due to an itchy nose*** 0= No symptoms. 1=Mild symptoms (Symptoms are easily tolerated). 2= Moderate symptoms (Intense symptoms but tolerable). 3= Hard symptoms (Symptoms are hard to tolerate, effects daily activities and/or sleep).

0

- 1
- 2
- 3

**Estimate the discomfort during the last 24 hours due to sneezinggar?*** 0= No symptoms. 1=Mild symptoms (Symptoms are easily tolerated). 2= Moderate symptoms (Intense symptoms but tolerable). 3= Hard symptoms (Symptoms are hard to tolerate, effects daily activities and/or sleep).

0

- 1
- 2
- 3

**Estimate the discomfort during the last 24 hours due to a blocked nose.*** 0= No symptoms. 1=Mild symptoms (Symptoms are easily tolerated). 2= Moderate symptoms (Intense symptoms but tolerable). 3= Hard symptoms (Symptoms are hard to tolerate, effects daily activities and/or sleep).

0

- 1
- 2
- 3

**Estimate the discomfort during the last 24 hours due to red and/or itchy eyes.*** 0= No symptoms. 1=Mild symptoms (Symptoms are easily tolerated). 2= Moderate symptoms (Intense symptoms but tolerable). 3= Hard symptoms (Symptoms are hard to tolerate, effects daily activities and/or sleep).

0

- 1
- 2
- 3

**Estimate the discomfort during the last 24 hours due to runny eyes*** 0= No symptoms. 1=Mild symptoms (Symptoms are easily tolerated). 2= Moderate symptoms (Intense symptoms but tolerable). 3= Hard symptoms (Symptoms are hard to tolerate, effects daily activities and/or sleep).

0

- 1
- 2
- 3

**Estimate the discomfort during the last 24 hours due to coughing*** 0= No symptoms. 1=Mild symptoms (Symptoms are easily tolerated). 2= Moderate symptoms (Intense symptoms but tolerable). 3= Hard symptoms (Symptoms are hard to tolerate, effects daily activities and/or sleep)

0

- 1
- 2
- 3

**Estimate the discomfort during the last 24 hours due to heavy breathing or veezing.*** 0= No symptoms. 1=Mild symptoms (Symptoms are easily tolerated). 2= Moderate symptoms (Intense symptoms but tolerable). 3= Hard symptoms (Symptoms are hard to tolerate, effects daily activities and/or sleep)

0

- 1
- 2
- 3

**Have you used antihistamine tablets during the past 24 hours?*Obligatorisk**(e.g. Cetirizin, Loratadin, Aerius).

- Yes
- No

**Have you used antihistamine eye drops during the past 24 hours?* (((**(e.g Opatanol, Emadine).

- Yes
- No

**Have you used cortisone nasalspray during the past 24 hours?Obligatorisk**(e.g Nasonex, Avamys.)

- Yes
- No

**Have you used broncho dialating medication during the past 24 hours?*Obligatorisk(**(e.g. Bricanyl, Ventoline, Symbicort).

- Yes
- No
