# Supplementary figures and images for: Limited beneficial effects of systemic steroids when added to standard of care treatment of seasonal allergic rhinitis
Source: Sci Rep. 2023 Nov 10;13:19649. doi: 10.1038/s41598-023-46869-4 (PMC10638382; doi:10.1038/s41598-023-46869-4)

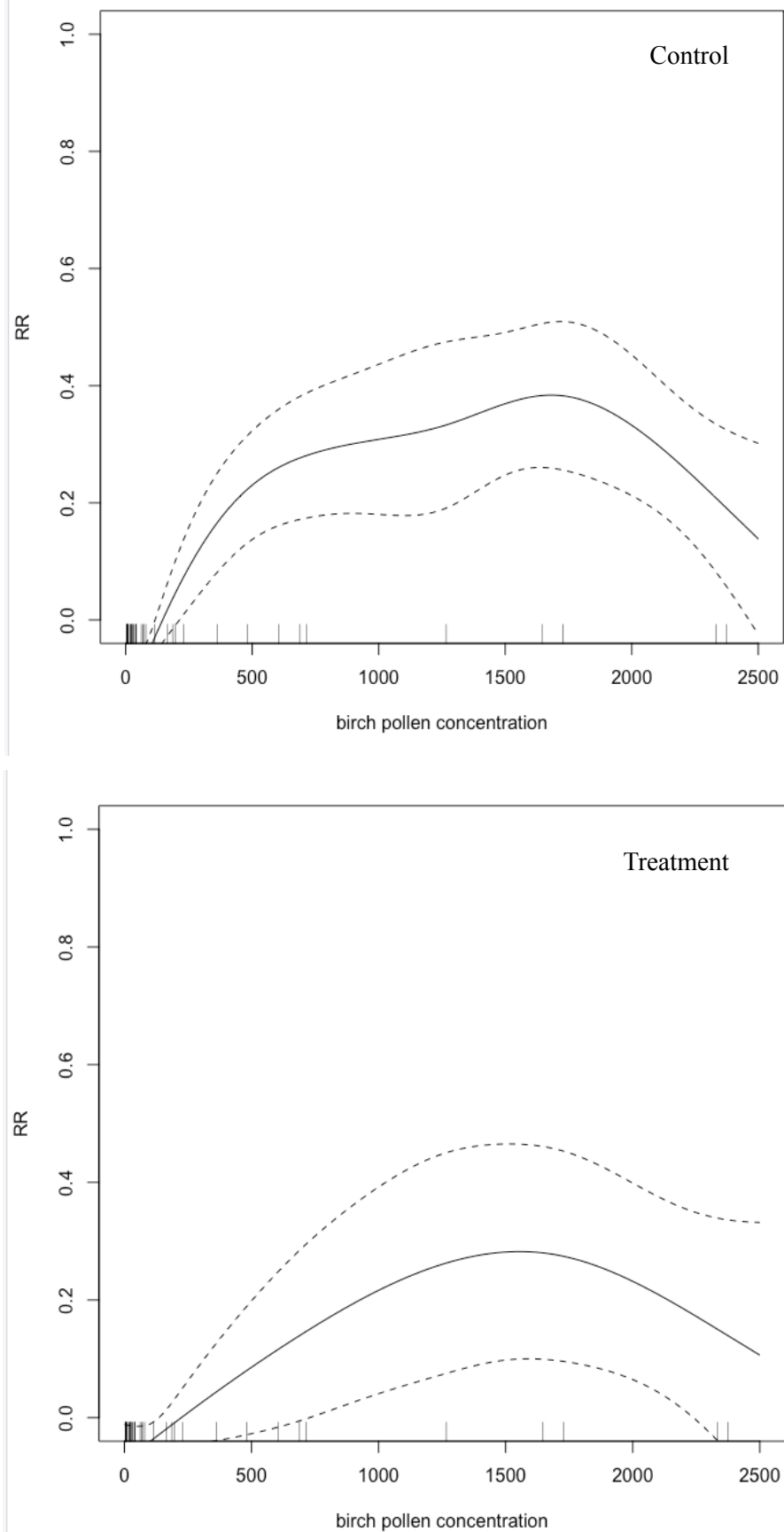

Supplement Fig 1. The relative risk of symptoms at different pollen concentrations.

Supplement: Supplementary file 3 — Supplementary Figure 1. [file 41598_2023_46869_MOESM3_ESM.pdf]
