# Supplementary material for: Limited beneficial effects of systemic steroids when added to standard of care treatment of seasonal allergic rhinitis
Source: Sci Rep. 2023 Nov 10;13:19649. doi: 10.1038/s41598-023-46869-4 (PMC10638382; doi:10.1038/s41598-023-46869-4)
Supplement: Supplementary file 4 — Supplementary Figure 2. [file 41598_2023_46869_MOESM4_ESM.docx]

**Supplement Figure 2.** Logarithmic scale of pollen count during the spring of 2019.
