# Supplementary material for: Limited beneficial effects of systemic steroids when added to standard of care treatment of seasonal allergic rhinitis
Source: Sci Rep. 2023 Nov 10;13:19649. doi: 10.1038/s41598-023-46869-4 (PMC10638382; doi:10.1038/s41598-023-46869-4)
Supplement: Supplementary file 5 — Supplementary Tables. [file 41598_2023_46869_MOESM5_ESM.docx]

Blood samples

**Table 1 supplement.** Blood samples before- (pre) and after (post) treatment with placebo.

|  |  |  |  |  |  |  |
| --- | --- | --- | --- | --- | --- | --- |
| **Placebo** | pre ACTH  (1,5-14) | Post ACTH  (1,5-14) | pre Kortisol  (ref interval*) | post Kortisol  (ref interval*) | pre CTx  (ref interval**) | post CTx  (ref interval**) |
| P-1 | 1,8 | 2,7 | 367 | 386 | 131 | 185 |
| P-2 | 3,7 | 3,5 | 265 | 387 | 114 | 424 |
| P-3 | 2,6 | 1,6 | 273 | 260 | 33 | 45 |
| P-4 | 2,2 | 3,6 | 226 | 258 | 462 | 619 |
| P-5 | 2,5 | 2,6 | 171 | 235 | 219 | 522 |
| P-6 | 4,5 | 5,1 | 260 | 220 |  | 165 |
| P-7 | 5,9 | 6 | 202 | 266 |  | 355 |
| P-8 | 4,8 | 14 | 240 | 748 |  | 527 |
| P-9 | 5 | 5,2 | 198 | 150 |  | 128 |
| P-10 | 2,8 | 2,7 | 190 | 188 |  | 135 |
| P-11 |  |  |  |  |  |  |
| P-12 | 5,1 | 4,4 | 201 | 234 | 530 | 491 |
| P-13 | 5 | 5,3 | 142 | 187 | 172 | 172 |
| P-14 | 3 | 4,3 | 322 | 411 | 165 | 197 |
| P-15 | 1,6 | 2 | 236 | 297 | 100 | 124 |
| P-16 | 2,8 | 3 | 303 | 147 | 434 | 52 |
| P-17 | 3,5 | 4,8 | 289 | 319 | 166 | 513 |
| P-18 | 4,8 | 6,4 | 146 | 216 |  | 187 |
| P-19 | 3,6 | 4 | 149 | 234 | 48 | 33 |
| P-20 | 3,1 | 2,1 | 136 | 251 | 50 | 101 |
| P-21 | 6,5 | 6,2 | 279 | 170 | 166 | 266 |
| **Mean** | **3,7** | **4,5** | **229,8** | **278,2** | **199,3** | **262,1** |

* Reference interval cortisol 133-537 nmol/L (06.00-10.00 a.m.) and 68-327 nmol/L 16.00-20.00 p.m)

** Reference interval CTx pre-menopausal women < 640 ng/L, post-menopausal women < 1040 ng/L, men < 730 ng/L

**Table 2 supplement.** Blood samples before- (pre) and after (post) treatment with Depo-Medrol.

|  | |  |  |  |  |  |
| --- | --- | --- | --- | --- | --- | --- |
| **Treatment** | pre ACTH  (1,5-14) | Post ACTH  (1,5-14) | pre Kortisol  (ref interval*) | post Kortisol  (ref interval*) | pre CTx  (ref interval**) | post CTx  (ref interval**) |
| T-1 | 7,7 | 5,6 | 415 | 317 | 314 | 256 |
| T-2 | 13 | 5,3 | 149 | 261 | 106 | 193 |
| T-3 | 6,3 | 5,8 | 265 | 224 | 64 | 82 |
| T-4 | 13 | 6,4 | 227 | 137 | 233 | 209 |
| T-5 | 4,5 |  | 221 |  |  |  |
| T-6 | 3,6 | 3,1 | 367 | 249 | 159 | 72 |
| T-7 | 5,3 | 4,3 | 256 | 255 |  | 517 |
| T-8 | 2,8 | 3,1 | 183 | 186 |  | 107 |
| T-9 | 2,3 | 1,7 | 186 | 123 |  | 247 |
| T-10 | 4,5 | 2,6 | 298 | 233 |  | 48 |
| T-11 | 2,5 | 3,2 | 188 | 184 |  |  |
| T-12 | 2,2 | 3,4 | 283 | 461 | 213 | 333 |
| T-13 | 4,2 | 3,5 | 166 | 164 |  |  |
| T-14 | 2,8 | 2,5 | 177 | 227 |  |  |
| T-15 | 7,1 | 7 | 285 | 304 | 443 | 322 |
| T-16 | 5 | 7,6 | 217 | 349 | 169 | 387 |
| T-17 | 8,9 | 5,7 | 375 | 334 | 33 | 41 |
| T-18 | 4,1 | 5,3 | 212 | 278 |  | 224 |
| T-19 | 2 | 2,3 | 595 | 376 | 318 | 174 |
| T-20 | 5,7 | 7,6 | 244 | 351 | 108 | 200 |
| T-21 | 3,6 | 3,5 | 273 | 268 | 186 | 172 |
| **Mean** | **5,3** | **4,5** | **265,8** | **264,1** | **195,5** | **210,8** |

* Reference interval cortisol 133-537 nmol/L (06.00-10.00 a.m.) and 68-327 nmol/L 16.00-20.00 p.m

** Reference interval CTx pre-menopausal women < 640 ng/L, post-menopausal women < 1040 ng/L, men < 730 ng/L
